# Functional Connectivity Alterations and Molecular Characterization of the Anterior Cingulate Cortex in Tinnitus Pathology without Hearing Loss

**DOI:** 10.1002/advs.202304709

**Published:** 2023-11-27

**Authors:** Ting Fan, Peng‐Fei Guan, Xiao‐Fang Zhong, Meng‐Ya Xiang, Ying‐Qiu Peng, Ruo‐Qiao Zhou, Jia‐Min Gong, Yu‐Qing Zheng, A‐Qiang Dai, Jia‐Ling Feng, Hong‐Zhe Yu, Jian Li, Hua‐Wei Li, Yun‐Feng Wang

**Affiliations:** ^1^ ENT Institute and Department of Otorhinolaryngology EYE & ENT Hospital Fudan University Shanghai 200031 China; ^2^ NHC Key Laboratory of Hearing Medicine Fudan University Shanghai 200031 China; ^3^ Clinical Laboratory Center Children's Hospital of Fudan University Shanghai 201102 China; ^4^ Zhejiang Chinese Medical University Hangzhou Zhejiang 310053 China

**Keywords:** anterior cingulate cortex, auditory cortex, electroencephalography, glutamatergic synapse, tinnitus

## Abstract

Compared with individuals with hearing loss, tinnitus patients without hearing loss have more psychological or emotional problems. Tinnitus is closely associated to abnormal metabolism and function of the limbic system, a key brain region for emotion experience, but the underlying molecular mechanism remains unknown. Using whole‐brain microvasculature dynamics imaging, the anterior cingulate cortex (ACC) is identified as a key brain region of limbic system involve in the onset of salicylate‐induced tinnitus in mice. In the tinnitus group, there is enhanced purine metabolism, oxidative phosphorylation, and a distinct pattern of phosphorylation in glutamatergic synaptic pathway according to the metabolome profiles, quantitative proteomic, and phosphoproteomic data of mice ACC tissue. Electroencephalogram in tinnitus patients with normal hearing thresholds show that the functional connectivity between pregenual anterior cingulate cortex and the primary auditory cortex is significantly increased for high‐gamma frequency band, which is positively correlated with the serum glutamate level. These findings indicate that ACC plays an important role in the pathophysiology of tinnitus by interacting with the primary auditory cortex and provide potential molecular targets in the ACC for tinnitus treatment.

## Graphical abstract

Anterior cingulate cortex (ACC) is a brain region of limbic system. There is enhanced metabolism and a distinct pattern of phosphorylation in glutamatergic synaptic pathway in ACC tissue of tinnitus mice. Electroencephalogram in tinnitus patients show increased functional connectivity between ACC and the primary auditory cortex. These findings indicate that ACC involves in the pathophysiology of tinnitus by interacting with the primary auditory cortex.

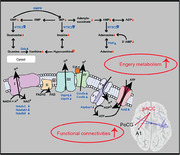

## 1 Introduction

Tinnitus is a hallucination featured by hearing sound without relevant objective exoteric stimulation,^[^ ^1^ ^]^ with a prevalence of ≈14.4% in adults and 32.0% in the elderly.^[^ ^2^, ^3^ ^]^ Bothersome tinnitus can further lead to mood disorders like depression and anxiety.^[^ ^4^ ^]^ Although several approaches for alleviating tinnitus exist, there is no effective curing method yet. To find possible therapy targets, the mechanism of tinnitus has long been one of the major topics in hearing research. Because tinnitus is the most common pathology associated with hearing loss, it is commonly assumed that tinnitus is caused by peripheral hearing loss followed by variations in the central auditory pathways. However, tinnitus can also present without hearing loss clinically. Approximately 4.7–46% of children with normal audiograms were estimated to have tinnitus.^[^ ^5^, ^6^ ^]^ The pathogenesis for these patients remains largely unknown now.

One view is that tinnitus in cases without hearing loss might be more related to psychological or emotional problems.^[^ ^7^ ^]^ Previous studies reported higher prevalence of depression in tinnitus patients withnormal hearing thresholds compared with the whole tinnitus population (41.7% vs 25.6%).^[^ ^4^, ^8^ ^]^ These findings led some researchers to propose that the limbic system,which provides the substrate for emotion experience, may play important roles in tinnitus perception.^[^ ^9^ ^]^ In fact, tinnitus patients without hearing loss have shown significant alterations in white matter microstructures in corpus callosum and cingulum, both of which are limbic structures in the brain.^[^ ^10^ ^]^ Many animal experiments also identified tinnitus‐related differences in structures of the limbic system.^[^ ^11^, ^12^ ^]^ However, these studies typically use neurophysiological or functional imaging techniques, the molecular mechanism underlying the abnormal psychological functioning of the limbic system has yet to be elucidated. Moreover, studies that implicated limbic involvement report disparate sites, including anterior cingulate cortex (ACC),^[^ ^11^ ^]^ amygdala,^[^ ^12^ ^]^ hippocampus,^[^ ^12^ ^]^ nucleus accumbens,^[^ ^13^ ^]^ and so on. Thus, the exact nature of limbic system involvement in tinnitus without hearing loss is far from clear.

Sodium salicylate, a formerly used antirheumatic agent sharing the same active compound with anti‐inflammatory drug aspirin, can induce the adverse reaction of acute tinnitus at high doses,^[^ ^14^ ^]^ and thus has been widely adopted in the establishments of animal models with the purpose of studying the mechanisms of tinnitus.^[^ ^11^, ^15^, ^16^, ^17^ ^]^ Furthermore, the plasm concentration of salicylate could reach the peak value about five minutes after salicylate administration and maintain about six hours,^[^ ^18^ ^]^ making it possible to continuously monitor the brain activation mapping before and after salicylate treatment. In this study, we measured the whole‐brain microvasculature dynamics after salicylate treatment in mice using functional ultrasound (fUS)^[^ ^19^ ^]^ for the first time, to determine the brain structure in the limbic system that was most affected by tinnitus.

High throughput omics technologies provide comprehensive measurements of biological molecules and profound insights into pathological phenotypes, thus have been used to investigate the mechanisms of neurological disorders, including but not limited to autism,^[^ ^20^ ^]^ Alzheimer's disease,^[^ ^21^, ^22^ ^]^ and posttraumatic stress disorder.^[^ ^23^ ^]^ Previous studies have identified plasma metabolomic biomarkers for tinnitus,^[^ ^24^, ^25^ ^]^ but to our knowledge, the metabolome and proteome profiles in brain tissue are lacking. Moreover, the brain is one of the organs with the highest levels of phosphorylation modifications because of its unique cellular function and specialized signaling networks.^[^ ^26^ ^]^ In order to gain valuable insights into a disorder as complex as tinnitus, we examined tinnitus‐related metabolomics, proteomics, and protein phosphorylation modification characteristics of the targeted limbic region in this study.

High‐density electroencephalography (EEG), which was frequently used to study central processing in tinnitus, provides the advantages of high temporal resolution, time efficiency, and noise avoidance.^[^ ^27^ ^]^ Finally, we investigated potential correlations between the metabolic markers and limbic system activity in tinnitus patients without hearing loss using EEG examination.

## 2 Results

### 2.1 Identification of ACC as the Most Activated Brain Region in the Limbic System During Tinnitus Onset

**Figure 1:**
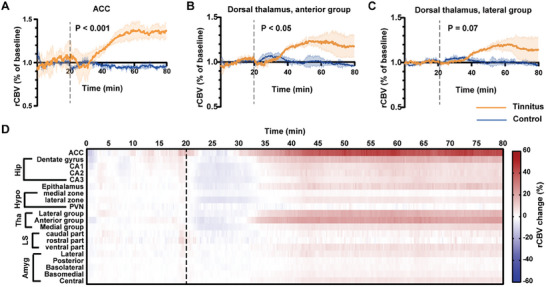
Precise mapping of sodium salicylate impact on local cerebral blood volume of the limbic system. A–C) Relative cerebral blood volume (rCBV) changes of different brain areas in tinnitus (n = 4) and control (n = 3) mice. The *p*‐value was calculated using two‐way ANOVA. D) Time‐dependent effects after sodium salicylate challenge. rCBV change = (rCBV of tinnitus group – rCBV of control group) / rCBV of control group * 100%. The dashed line means injection time. ACC: anterior cingulate cortex; Hip: Hippocampus; CA1, CA2, CA3: cornu ammonis areas; Hypo: hypothalamus; PVN: paraventricular nucleus of hypothalamus; Tha: Thalamus; LS: Lateral septum; Amyg: Amygdala.

In the present study, tinnitus was induced 1 hour after the first dose of salicylate in 96% (25/26) of the salicylate‐treated mice (Figure S1, Supporting Information), suggesting that salicylate administration was able to induce tinnitus in mice in a very short time. We focus on the limbic system. Compared with the control group, the relative local cerebral blood volume (rCBV) in tinnitus group increased by 47.37%, 25.65%, and 21.06% in ACC, anterior group of the dorsal thalamus, and lateral group of the dorsal thalamus in max, respectively (**Figure** **1**A–C). There was no significant difference of rCBV between tinnitus and control groups in other limbic structures (Figure S2, Supporting Information). The time‐dependent activation of the limbic system was summed up in Figure 1D. Compared with the control group, the rCBV of ACC in the tinnitus group increased 10—15 min after salicylate administration, and could be maintained to at least 1 hour. The activation maps of ACC were shown in Figure S3 (Supporting Information).

### 2.2 Metabolomic Profiling of the ACC in Tinnitus Mice

**Figure 2:**
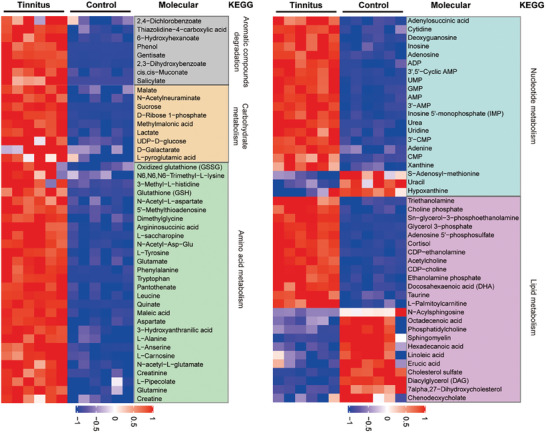
Differential metabolites of the anterior cingulate cortex samples in tinnitus and control mice. Metabolites are classified by Kyoto Encyclopedia of Genes and Genomes (KEGG) metabolic pathways. Anterior cingulate cortex tissue from 25 tinnitus mice and 28 healthy control mice were combined into 6 samples in each group.

In this study, we identified 132 differentially expressed metabolites in ACC between tinnitus and control groups, based on the variable importance in the projection value > 1 in the robust orthogonal partial least squares‐discriminant analysis (OPLS‐DA) and *p* < 0.05 in the student's *t*‐test (source data of all identified metabolites in Data S1, Supporting Information). The metabolomics profiling reveals a high metabolite endotype characterized by higher level of carbohydrates, amino acids, and nucleotides (**Figure** **2**). This finding is in agreement with the fUS results, suggesting increased brain activity in the ACC under tinnitus situation.

### 2.3 Increased Purine Metabolism and Oxidative Phosphorylation in ACC of Tinnitus Mice

**Figure 3:**
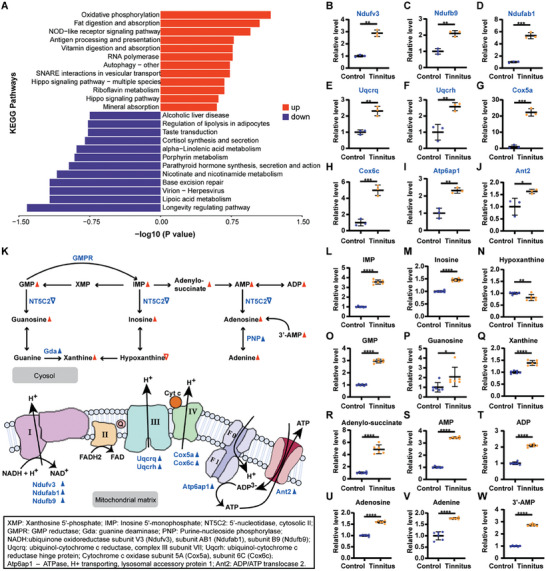
Purine metabolism and oxidative phosphorylation in ACC of tinnitus mice. A) Significantly down and up‐regulated pathways identified by KEGG pathway analysis. B–J) Relative abundance of the subunits of representative enzymes (ubiquinone oxidoreductase, ubiquinol‐cytochrome C reductase, Cytochrome C oxidase and ATPase) involved in oxidative phosphorylation. ACC tissue from 25 tinnitus mice and 28 healthy control mice were combined into 3 samples in each group. K) Schematic diagram of purine metabolism and oxidative phosphorylation during tinnitus, derived from metabolomics and proteomics. Metabolites increased or decreased in ACC of tinnitus group are denoted by red filled or empty triangles. The blue filled and empty triangles indicate up and down‐regulated metabolic enzymes, respectively. L–W) Relative levels of metabolites related to purine metabolism in ACC of tinnitus and control groups. ACC tissue from 25 tinnitus mice and 28 healthy control mice were combined into 6 samples in each group. Error bars indicate SD. ∗*p* < 0.05, ∗∗*p* < 0.01, ∗∗∗*p* < 0.001, ****p < 0.0001, two‐tailed *t*‐test.

A total of 3981 proteins were quantified (with the false discovery rate of 0.01, source data in Data S2, Supporting Information), and 745 proteins with differential levels were identified (adjusted *p*‐value < 0.05). Oxidative phosphorylation is the most significantly up‐regulated KEGG pathway in the tinnitus group (**Figure** **3**A). There was a significant up‐regulation of the enzyme subunits involved in oxidative phosphorylation, including Ndufv3, Ndufb9, Ndufab1, Uqcrq, Uqcrh, Cox5a, Cox6c, and Atp6ap1 (Figure 3B–I). Additionally, the expression level of ADP/ATP translocase2 (Ant2) in tinnitus group was 1.63‐fold of the control group (Figure 3J), which is responsible for ADP/ATP transportation across the mitochondrial inner membrane. Combined analysis of the metabolomics and proteomics data showed that purine metabolism is one of the top 10 up‐regulated KEGG pathways in the tinnitus group. Eleven purine‐related metabolites were significantly increased in the tinnitus group, including AMP and ADP (Figure 3L–W). Taken together, our results indicate that upregulated purine metabolism during tinnitus increased the ADP level in cytoplasm, which can be transported into mitochondrial matrix and used as the substrate of oxidative phosphorylation to synthetize more ATP for the abnormal neural activities in ACC (Figure 3K).

### 2.4 Phosphoproteomic Profiling of the ACC in Tinnitus Mice

**Figure 4:**
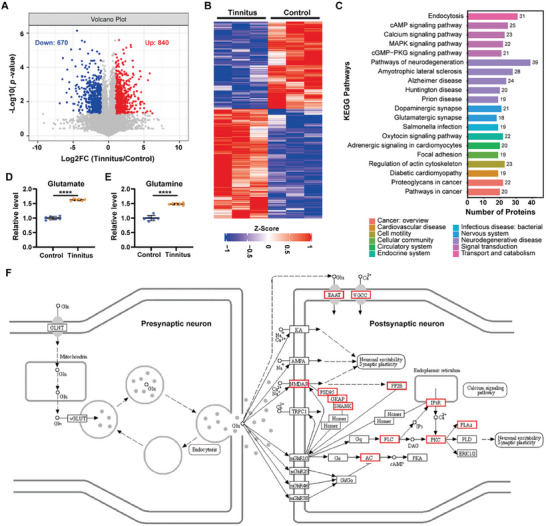
Phosphoproteomic profiling of tinnitus and control group. A) Volcano plot displaying the up‐ and down‐ regulated phosphosites in the tinnitus group. B) Heatmap of phosphoproteins corresponding to phosphopeptides whose expression are significantly different in tinnitus and control group. C)Top 20 differential KEGG pathways according to KEEG enrichment analysis. D,E) Relative abundance of glutamate and glutamine between tinnitus and control group. ACC tissue from 25 tinnitus mice and 28 healthy control mice were combined into 6 samples in each group. F) KEGG database glutamatergic synapse pathway connected with phosphoproteomic profile of tinnitus group, in which the red circle represents the protein with significant difference in phosphorylation regulation of tinnitus group.

In this study, a total of 14 430 phosphosites, 7443 phosphopeptides, and 2591 phosphoproteins were detected (Data S3, Supporting Information). Among the identified phosphorylation sites, 80.58% were Ser, followed by Thr (18.05%) and Tyr (1.37%), which was similar to previous studies.^[^ ^28^ ^]^ After intercalating the cutoff fold change to greater than 2 and adjusted *p*‐value < 0.05, we identified 840 upregulated phosphosites and 670 downregulated phosphosites in the tinnitus group, compared to the control group (**Figure** **4**A). The corresponding phosphoproteins of these differential phosphopeptides could distinguish tinnitus group from the control (Figure 4B). The top 20 differential KEGG pathways are shown in Figure 4C. Notably, we found different phosphorylation patterns in the glutamatergic synapse pathway, mainly in the postsynaptic elements, including glutamate receptors, glutamate transporters, postsynaptic density components, and several intracellular signal transduction molecules (Figure 4F). Increased glutamatergic activity in ACC appears to be associated to the sense of tinnitus, as both glutamate and glutamine were significantly up‐regulated in the ACC of tinnitus mice (Figure 4D,E).

### 2.5 Electroencephalography Showed Increased Functional Connectivity of ACC in Tinnitus Patients without Hearing Loss

**Figure 5:**
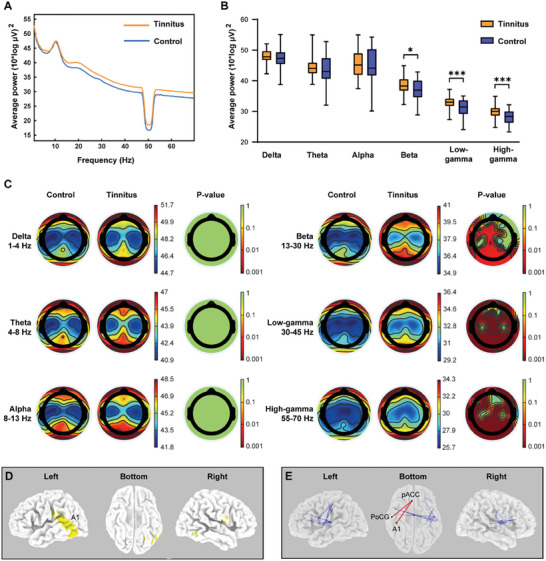
Significantly increased EEG source and functional connectivity for high‐gamma frequency band in tinnitus patients without hearing loss. A) Comparison of whole‐brain power spectrum between tinnitus (n = 64) and control (n = 44) groups. B) Comparisons of average EEG power at six frequency bands between the tinnitus and control groups. ∗*p* < 0.05, ∗∗*p* < 0.01, ∗∗∗*p* < 0.001, two‐tailed Mann‐Whitney *U* test. C) Topographic maps of the average oscillatory power spectrum over different frequency bands for the tinnitus and control groups. D) Significantly increased EEG source for high‐gamma frequency band in tinnitus group compared with the control group. The locations of significant cluster are shown with yellow color. E) Significantly increased lagged nonlinear connectivity in high‐gamma frequency band in tinnitus group compared with the control group. The significant connectivity wires between primary auditory cortex (A1), postcentral gyrus (PoCG) and pregenual anterior cingulate cortex (pgACC) are shown with red color in the bottom view.

Resting‐state EEG was conducted in 64 tinnitus patients with normal hearing thresholds and 44 health control subjects. The results showed that tinnitus had significant effects on the power spectra between 15–70 Hz (**Figure** **5**A). Tinnitus patients without hearing loss showed significant increases in the current density of high frequencies as compared to healthy control subjects, including beta, low‐gamma, and high‐gamma frequency (Figure 5B). Figure 5C shows the topographical maps of the average oscillatory power spectrum across six frequency bands. The temporoparietal areas, which are closely associated with auditory processing and cognitive perception,^[^ ^29^ ^]^ were the predominant location of the increased spectral power of beta and gamma frequencies in tinnitus patients without hearing loss, including the auditory cortex, precentral and postcentral cortex, middle cingulate cortex, and insula. Source localization analysis revealed that tinnitus patients had significantly higher brain activity in high‐gamma frequency in the primary auditory cortex (Figure 5D). The average EEG current density of the primary auditory cortex was 13.72 (10*log µm)^2^ and 8.79 (10*log µm)^2^ in tinnitus patients and healthy individuals, respectively.

Source localization analysis showed that for high‐gamma frequency band, tinnitus patients had significantly increased lagged nonlinear connectivity among 12 pairs of brain areas (Table S1, Supporting Information). It is worth noting that significantly increased lagged phase synchronization was found between primary auditory cortex (A1) and pregenual anterior cingulate cortex (pgACC) for tinnitus patients in comparison to healthy subjects (0.039 versus 0.030, Figure 5E), suggesting that ACC, as one of the important structures in limbic system, may be closely associated with auditory perception and involved in the process of tinnitus. The functional connectivity between the postcentral gyrus (PoCG) and pgACC was also significantly increased (Figure 5E), indicating that ACC is also involved in somatosensory regulation in tinnitus patients. The lagged phase synchronization was 0.034 and 0.028 in tinnitus patients and healthy individuals, respectively. No significant connectivity change was found in delta, theta, alpha, beta, or low‐gamma frequency band.

### 2.6 Correlation of Serum Glutamate Level and Functional Connectivity of ACC in Tinnitus Patients without Hearing Loss

**Figure 6:**
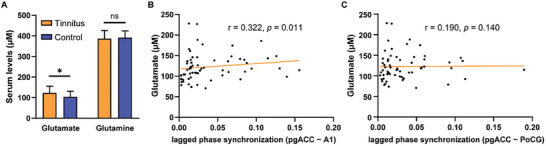
Correlations between glutamate serum level and ACC functional connectivity in tinnitus patients without hearing loss. A) Serum levels of glutamate and glutamine in tinnitus (n = 62) and control (n = 23) groups. ∗*p* < 0.05, ns: no significant difference, two‐tailed *t*‐test. B) Correlation between glutamate serum level and lagged phase synchronization between pgACC and A1 in tinnitus patients. C) Correlation between glutamate serum level and lagged phase synchronization between pgACC and PoCG in tinnitus patients. Correlation analysis was performed by Spearman's Rho.

Serum glutamate and glutamine concentrations were analyzed in tinnitus patients without hearing loss and healthy control participants. A significantly higher mean serum glutamate level was found in tinnitus patients as compared to healthy controls (122.91 ± 32.88 vs 103.92 ± 26.98 µM, **Figure** **6**A). There was no significant difference of the glutamine levels between two groups (386.70 ± 39.80 vs 391.89 ± 32.65 µM, Figure 6A). To reveal if glutamate serum level could reflect abnormal ACC activities in tinnitus patients, correlation analysis was performed to investigate the relationship between glutamate concentrations and EEG properties, which is shown in Figure 6B,C. Notably, there is significantly positive correlation between glutamate concentrations and the lagged phase synchronization of pgACC and A1 (r = 0.322, *p* = 0.011). No significant association was observed between glutamate serum level and Tinnitus Handicap Inventory or Visual Analogue Scale scores in tinnitus patients without hearing loss.

## 3 Discussion

The limbic system has been proved to be involved in tinnitus pathology and suggested as an attractive target region for tinnitus treatment.^[^ ^13^ ^]^ However, previous studies have reported a complex network involved multiple limbic regions, making it difficult to tell which part of the changes in these regions account for tinnitus perception instead of emotional experience, memory and spatial awareness, or multi‐sensory integration.^[^ ^30^ ^]^ Herein, we found significantly increased neural activities in ACC after salicylate administration in mice and identified ACC as the key limbic structure during the development of tinnitus. Increased purine metabolism, oxidative phosphorylation, and glutamate level were found in ACC tissue of tinnitus mice, which may be responsible for the abnormal activity of ACC during salicylate‐induced tinnitus. The EEG data showed that the functional connectivity between ACC and the primary auditory cortex enhanced in tinnitus patients without hearing loss, which was positively correlated with the serum glutamate level. Taken together, our study indicated that ACC was the key structure in the limbic system in the pathology of tinnitus, probably via interaction of the auditory cortex through glutamatergic synapses.

In this study, 96% of the mice treated with salicylate develops tinnitus, suggesting that salicylate is a reliable inducer of tinnitus. However, as salicylate has been shown to consistently cause both tinnitus and hyperacusis,^[^ ^30^, ^31^ ^]^ it is crucial to isolate the neural mechanisms associated with tinnitus from hyperacusis when using salicylate model. Sound‐evoked hyperactivity of the auditory system is the main characteristic of hyperacusis. For example, a significant increase in sound‐evoked activity in the auditory cortex was observed with local infusion of salicylate into the amygdala,^[^ ^31^ ^]^ suggesting that amygdala was predominantly responsible for salicylate‐induced hyperacusis. In this study, the fUS examination in quiet environment showed that the administration of salicylate activated ACC but not amygdala. Moreover, Fan X et al. found that ACC stimulation could modulate the spontaneous firing rate of the auditory cortex, which is a key feature of tinnitus.^[^ ^11^ ^]^ Therefore, we believed that the neural and biological changes of ACC observed in this study was primarily related to tinnitus. It should also be noted that salicylate could result in mild hearing loss.^[^ ^31^ ^]^ Extension of the changes we found using salicylate model to the mechanism of tinnitus without hearing loss should be treated with caution.

We firstly characterized the global metabolic and protein patterns of ACC in salicylate‐induced tinnitus mice. Brain activity is extremely dependent on oxidative phosphorylation to provide energy.^[^ ^32^ ^]^ The oxidative phosphorylation system consists of mitochondrial inner membrane electron transport chain (complexes I to IV) and ATP synthase (complex V). Specifically, we found that the expression level of Cox5a increased ≈22 folds in the ACC of tinnitus mice compared with healthy controls. Cox5a is a subunit of cytochrome c oxidase (complex IV), which is essential in the assembly and regulation of mitochondrial respiratory chain holoenzyme.^[^ ^33^, ^34^ ^]^ The activity of mitochondrial complex IV was dramatically decreased in Alzheimer's disease as an early alteration of brain bioenergetic dysfunction.^[^ ^35^, ^36^ ^]^ We detected increased Cox5a after 5 days of salicylate treatment in tinnitus mice, indicating that Cox5a may serve as an early‐stage index to reflex ATP generation and neural activity in ACC.

One of the cellular mechanisms enabling neuronal hyperactivity in tinnitus has been attributed to the up‐regulation of excitatory neurotransmitter. Increased glutamate level has been found in the dorsal cochlear nucleus, primary auditory cortex,^[^ ^37^ ^]^ and ACC in the present study. Furthermore, we observed a different phosphorylation modification pattern in the glutamatergic synapse pathway in the ACC, suggesting that glutamate mediated signal transduction was closely related to tinnitus pathology. Treatment with N‐methyl‐D‐aspartic acid (NMDA) receptor blocker on auditory cortex could attenuate tinnitus in animals.^[^ ^38^ ^]^ However, blockage of the NMDA channel on the ACC level has not been reported before. Considering that ACC is involved in both auditory and emotional regulation, treatment targeting the ACC may lead to both tinnitus relief and emotion improvement in the future.

Increased gamma activity has been associated with firing synchronization in cortical regions^[^ ^39^, ^40^ ^]^ and enhanced synchronous gain,^[^ ^41^ ^]^ and thus been linked to tinnitus. The EEG data in this study showed increased functional connectivity for high‐gamma frequency band between pgACC and auditory cortex in tinnitus patients with normal hearing thresholds, which is consistent of the previous research.^[^ ^42^ ^]^ Because the lagged phase synchronization was unable to predict the causative nature of the interactions, it is not clear whether the enhanced connectivity indicates tinnitus‐provoking or tinnitus‐suppressing activity between pgACC and the auditory cortex. One hypothesis is that the limbic system could prevent unwanted noise signals from reaching conscious perception through feedback pathways.^[^ ^43^ ^]^ Therefore, a possible explanation could be that the pgACC attempts to inhibit tinnitus by increasing tinnitus‐provoking input, but not enough to reduce the hyperactivity of the auditory cortex. More research is required to determine the exact mechanisms underlying the connections between ACC and the auditory cortex. Additionally, we found increased functional connectivity for high‐gamma frequency band among 12 pairings of brain areas, primarily outside the auditory system. This finding may provide a mechanism through which tinnitus are linked to negative emotions or other disorders.

There are some limitations in this study. Although we could obtain reliable CBV changes in tinnitus mice using baseline correction and group comparison, anesthesia may affect the brain activities during fUS examination.^[^ ^44^ ^]^ Further experiments using awake mice could avoid the bias of anesthesia and provide stronger evidence. To carry out multi‐omics analyzing of the ACC, more than 50 mice were used to harvest ACC tissues. Increase of the metabolite coverage and decrease of the mice number utilized in analogous researches may come true when a specific extraction method and targeted chromatography column are used. In addition, considering the variety type of cells in the ACC area, single‐cell metabolomic analysis may be is a promising tool to profile more precise metabolites in the future. However, our results extended the molecular mechanism of salicylate‐induced tinnitus. Particularly, the identified changes in the ACC could enable discovery of ideal targets for tinnitus treatment in the future.

## 4 Experimental Section

### Human Participants

Sixty four subjects were recruited with chronic tinnitus (39.3± 12.6 years, 30 female and 34 male) and 44 subjects as health controls (34.0± 10.7 years, 26 female and 18 male) from Eye and ENT Hospital of Fudan University. There was no significant difference in age or gender distribution between tinnitus and control groups. Pure tone audiometry was conducted to exclude hearing impairment (Figure S4, Supporting Information). All the tinnitus patients had persistent tinnitus with a duration of more than 3 months. Detailed characteristics of the tinnitus patients were provided in Table S2 (Supporting Information). The experimental operations were approved by the Ethics Committee of Eye and ENT Hospital of Fudan University (2021167‐1), and the informed written consent from all participants was obtained prior to the research.

### Mice

C57BL/6J male mice aged 8‐week were purchased from Shanghai Jihui Laboratory Animal Care Co., Ltd. and used at 8–12 weeks of age. Mice were accommodated in a specific pathogen‐free facility in the Department of Laboratory Animal Science of Fudan University with free access to drinking water and chow (12‐h light/dark cycle). All animal research protocols were approved by Animal Welfare and Ethics Committee, Department of Laboratory Animal Science of Fudan University (202210021S).

### Development of Tinnitus Mice Model

In this study, salicylate‐induced tinnitus model was used and assessed the behavioral evidence of tinnitus with a reflex‐based gap detection method (Figure S1E–G, Supporting Information, details of the experimental methods can be found in supplementary materials). Animals that exhibit reduced ability to detect the silent gap in background noise were considered mice with tinnitus. Pre‐pulse inhibition test was used to exclude severe hearing loss and sensory gating dysfunction.

### Functional Ultrasound Imaging of The Mouse Brain

Before fUS imaging acquisition, mice were anesthetized with 1.5% isoflurane for initiation and then 0.5% isoflurane for maintenance. Mice were placed in the heating pat to maintain the body temperature at 37 °C. The skin of mice head was incised and clean the surface of skull thoroughly, which ensure undistorted propagation of ultrasound waves between probe and tissue. The brain fUS was continuously performed from 20 min before the first dose of salicylate injection to 1 hour after the injection. The control group was treated with the same amount of physiological saline.

All imaging sessions used fUS hardware and software (Iconeus, Paris, France) dedicated for small animals. Ultrasound data was acquired using ultrasound machine equipped with a linear ultrasound probe (128 elements, 15 MHz, Vermon; Tour, France) driven by an ultrafast ultrasound scanner (Aixplorer, Supersonic Imagine; Aix‐en‐Provence, France). The skull of the mouse was covered with isotonic coupling gel. The ultrasonic probe was aligned in the coronal plane 2 mm above the skull, repetitious scanning from Bregma 0.0 to Bregma 4.0 (step size: 0.2 mm).

The CBV of each brain region was calculated in lcoStudio software, and then normalized to the baseline (0 to 20 min) using a MATLAB script, setting up the measurement of the rCBV in different brain areas which occurred after injection of sodium salicylate or saline.

### Harvest of Mouse Anterior Cingulate Cortex Tissue

After all the behavioral testing, mice of tinnitus and control groups were killed by cervical dislocation. The brains were removed immediately and cut into fresh slices (500um per slice) on ice. Then tissues of ACC were dissected from brain slices, located from Bregma 1.42 mm, Interaural 5.22 mm to Bregma 0.22 mm, Interaural 3.58 mm according to The Mouse Brain in Stereotaxic Coordinate.^[^ ^45^ ^]^ In total, ACC samples from 25 tinnitus mice and 28 healthy control mice were harvested (≈15 mg tissue per sample). The processing methods of ACC samples before LC‐MS/MS analysis can be found in supplementary materials.

### Metabolomics

LC‐MS/MS analysis was performed using Ultra High Performance Liquid Chromatography (1290 Infinity LC, Agilent Technologies) coupled to a quadrupole time‐to‐flight (AB Sciex TripleTOF 6600). Metabolite identification and data preprocessing were conducted in XCMS (https://xcmsonline.scripps.edu). All processed data was analyzed by R package. OPLS‐DA was used to conduct multivariate data analysis, which showed clear separation of the tinnitus and control groups with the cross validated predictive ability of Q^2^ (cum) > 0.9 (Figure S5, Supporting Information). Robustness of the model was evaluated by the 7‐fold cross‐validation and response permutation testing.

### Proteomics and Phosphoproteomics

LC‐MS/MS analysis was performed on a timsTOF Pro mass spectrometry coupled to Nanoelute (Bruker Daltonics Inc., Germany). The parameters of mass spectrometer were set as Sun J et.al. describe before.^[^ ^46^ ^]^ The MaxQuant software (V1.6.14) was used for protein identification and quantitation. All differentially expressed proteins and phosphorylated proteins were queried and mapped to pathways based on the KEGG database. Enrichment analysis was applied using Fisher’ exact test followed by Benjamini‐Hochberg correction for multiple testing. R Version 3.5.1 was used to perform enrichment analysis and combine the KEGG annotation and enrichment result of the metabolomics and proteomics.

### Electroencephalography Recording and Data Analysis

EEG with 256 channels (EGI's HydroCel Geodesic Sensor Net) was applied to record resting‐state EEG data in human participants. The participants were asked to stay still, awake, close their eyes, and sit on the chair in a soundproof room. EEG was collected for ≈10 min using EGI Geodesic EEG Software (Net Station version 5.3). The impedance of all electrodes was controlled at less than 50 kΩ. The offline EEG data processing was conducted using MATLAB R2013b and EEGLAB toolbox. After data preprocessing, the Fourier transform was performed to obtain the spectra power of all scalp electrodes. The average power for the following frequency bands was calculated for further statistical analysis: delta (1–4 Hz), theta (4–8 Hz), alpha (8–13 Hz), beta (13–30 Hz), low‐gamma (30–45 Hz), and high‐gamma (55–70 Hz).

To explore the functional integration between spatially separated brain areas, source localization and functional connectivity analysis was conducted at six predefined frequency bands between all brain areas taken pair‐wise. Standardized low‐resolution brain electromagnetic tomography (sLORETA) was applied to evaluate the electrical activity of brain areas in six frequency bands for all participants. Electric neuronal activity for 47 Brodmann areas was computed by sLORETA as current density (A m^−2^).^[^ ^47^, ^48^ ^]^ The sLORETA was consist with a spatial space of 6239 voxels (voxel size 5*5*5 mm^3^), which defined by the digitized Montreal Neurological Institute 152 template.^[^ ^49^ ^]^ LORETA‐KEY was used to determine the locations of significant cluster. Nonparametric statistical analyses were performed by using functional sLORETA images (statistical non‐parametric mapping, SnPM) for each contrast. For each of the voxels in the region of interest, the built‐in voxel‐wise randomization tests of LORETA‐KEY were used to correct for multiple comparisons (5000 random permutations). Lagged phase synchronization,^[^ ^50^ ^]^ a nonlinear temporal synchrony measure, was used to explore the connection between the time‐varying signals recorded from two brain regions and measured by the sLORETA connectivity toolbox.

### Blood Samples and Biochemical Assays

Blood samples were taken from the human subjects who completed the EEG test. The serum was immediately separated and stored at −80 °C until analysis. High‐Performance Liquid Chromatography was used to assess the glutamate and glutamine levels in the serum. Based on retention times and peak areas, glutamate and glutamine were identified and quantified, and then compared to those found in external standards.

### Statistical Analysis

Mann‐Whitney *U* test, Student's *t* test, or Fisher’ exact test was adopted for between‐group comparisons, depending on the features of the data set. Benjamini‐Hochberg correction for multiple testing was applied to adjust *p*‐values when screening differentially expressed proteins and phosphosites. Spearman's Rho was used for correlation analysis between serum glutamate levels, clinical variables, and EEG indexes. The statistical analyses were conducted with IBM SPSS Statistics, v28 (IBM Corp., Armonk, NY) unless otherwise specified. The *p*‐value < 0.05 was considered to significant level for all statistical analyses in this study.

## Conflict of Interest

The authors declare no conflict of interest.

## Supporting information

Supporting Information

Supplementary Data S1

Supplementary Data S2

Supplementary Data S3

## Data Availability

The data that support the findings of this study are available in the supplementary material of this article.

## References

[1] D. Baguley , D. Mcferran , D. Hall , Tinnitus. Lancet 2013, 382, 1600. 23827090 10.1016/S0140-6736(13)60142-7

[2] C. M. Jarach , A. Lugo , M. Scala , P. A. Van Den Brandt , C. R. Cederroth , A. Odone , W. Garavello , W. Schlee , B. Langguth , S. Gallus , JAMA Neurol. 2022, 79, 888. 35939312 10.1001/jamaneurol.2022.2189 PMC9361184

[3] N. C. Chang , C. Y. Dai , W. Y. Lin , H. L. Yang , H. M. Wang , C. Y. Chien , K. Y. Ho , J. Int. Adv. Otol. 2019, 15, 99. 31058599 10.5152/iao.2019.6257 PMC6483451

[4] J. M. Bhatt , N. Bhattacharyya , H. W. Lin , Laryngoscope 2017, 127, 466. 27301552 10.1002/lary.26107 PMC5812676

[5] S. N. Rosing , J. H. Schmidt , N. Wedderkopp , D. M. Baguley , BMJ Open 2016, 6, e010596. 10.1136/bmjopen-2015-010596 PMC4893873 27259524

[6] S. N. Rosing , A. Kapandais , J. H. Schmidt , D. M. Baguley , Int. J. Pediatric. Otorhinolaryngol. 2016, 89, 112. 10.1016/j.ijporl.2016.07.036 27619040

[7] J. Choi , C. H. Lee , S. Y. Kim , Medicina 2021, 57, 114.33513909

[8] H. M. Kehrle , A. L. L. Sampaio , R. C. Granjeiro , T. S. De Oliveira , C. A. C. P. Oliveira , Rhinol. Laryngol. 2016, 125, 185. 10.1177/0003489415606445 26424781

[9] J. P. Rauschecker , A. M. Leaver , M. Mühlau , Neuron 2010, 66, 819. 20620868 10.1016/j.neuron.2010.04.032 PMC2904345

[10] Q. Chen , Z. Wang , H. Lv , P. Zhao , Z. Yang , S. Gong , Z. Wang , Front. Neurosci. 2020, 14, 591. 32612504 10.3389/fnins.2020.00591 PMC7308730

[11] X. Fan , Y. Song , Y. Du , J. Liu , S. Xiong , G. Zhao , M. Wang , J. Wang , F. Ma , L. Mao , Otol. Neurotol. 2021, 42, e1134. 33859133 10.1097/MAO.0000000000003183

[12] Y.‐C. Chen , X. Li , L. Liu , J. Wang , C.‐Q. Lu , M. Yang , Y. Jiao , F.‐C. Zang , K. Radziwon , G.‐D. Chen , W. Sun , V. P. Krishnan Muthaiah , R. Salvi , G.‐J. Teng , eLife 2015, 4, e06576. 25962854 10.7554/eLife.06576 PMC4426664

[13] A. M. Leaver , L. Renier , M. A. Chevillet , S. Morgan , H. J. Kim , J. P. Rauschecker , Neuron 2011, 69, 33. 21220097 10.1016/j.neuron.2010.12.002 PMC3092532

[14] Y. Cazals , Prog. Neurobiol. 2000, 62, 583. 10880852 10.1016/s0301-0082(00)00027-7

[15] P. Liu , D. Qin , X. Huang , H. Chen , W. Ye , X. Lin , J. Su , J. Comp. Physiol. A Neuroethol. Sens. Neural. Behav. Physiol. 2019, 205, 469. 31020389 10.1007/s00359-019-01339-z

[16] D. Qin , P. Liu , H. Chen , X. Huang , W. Ye , X. Lin , F. Wei , J. Su , Neurotox Res. 2019, 35, 838. 30820888 10.1007/s12640-019-0006-8

[17] J. Zugaib , C. C. Ceballos , R. M. Leão , Hear. Res. 2016, 332, 188. 26548740 10.1016/j.heares.2015.10.008

[18] G. A. Higgs , J. A. Salmon , B. Henderson , J. R. Vane , Proc. Natl. Acad. Sci. USA 1987, 84, 1417. 3103135 10.1073/pnas.84.5.1417 PMC304441

[19] C. Rabut , M. Correia , V. Finel , S. Pezet , M. Pernot , T. Deffieux , M. Tanter , Nat. Methods 2019, 16, 994. 31548704 10.1038/s41592-019-0572-y PMC6774790

[20] N. N. Parikshak , V. Swarup , T. G. Belgard , M. Irimia , G. Ramaswami , M. J. Gandal , C. Hartl , V. Leppa , L. D. L. T. Ubieta , J. Huang , J. K. Lowe , B. J. Blencowe , S. Horvath , D. H. Geschwind , Nature 2016, 540, 423. 27919067 10.1038/nature20612 PMC7102905

[21] E. C. B. Johnson , E. B. Dammer , D. M. Duong , L. Ping , M. Zhou , L. Yin , L. A. Higginbotham , A. Guajardo , B. White , J. C. Troncoso , M. Thambisetty , T. J. Montine , E. B. Lee , J. Q. Trojanowski , T. G. Beach , E. M. Reiman , V. Haroutunian , M. Wang , E. Schadt , B. Zhang , D. W. Dickson , N. Ertekin‐Taner , T. E. Golde , V. A. Petyuk , P. L. De Jager , D. A. Bennett , T. S. Wingo , S. Rangaraju , I. Hajjar , J. M. Shulman , et al., Nat. Med. 2020, 26, 769. 32284590 10.1038/s41591-020-0815-6 PMC7405761

[22] M. Wang , A. Li , M. Sekiya , N. D. Beckmann , X. Quan , N. Schrode , M. B. Fernando , A. Yu , L. Zhu , J. Cao , L. Lyu , E. Horgusluoglu , Q. Wang , L. Guo , Y.‐S. Wang , R. Neff , W.‐M. Song , E. Wang , Q. Shen , X. Zhou , C. Ming , S.‐M. Ho , S. Vatansever , H. Ü. Kaniskan , J. Jin , M.‐M. Zhou , K. Ando , L. Ho , P. A. Slesinger , Z. Yue , et al., Neuron 2021, 109, 257 . 33238137 10.1016/j.neuron.2020.11.002 PMC7855384

[23] D. L. Núñez‐Rios , J. J. Martínez‐Magaña , S. T. Nagamatsu , D. E. Andrade‐Brito , D. A. Forero , C. A. Orozco‐Castaño , J. L. Montalvo‐Ortiz , Biomedicines 2022, 10, 1107. 35625844 10.3390/biomedicines10051107 PMC9138536

[24] O. A. Zeleznik , D. B. Welling , K. Stankovic , L. Frueh , R. Balasubramanian , G. C. Curhan , S. G. Curhan , JAMA Otolaryngol.– Head Neck Surg. 2023, 149, 404. 36928544 10.1001/jamaoto.2023.0052 PMC10020935

[25] N. Ranjbar , A. Shahbazi , N. Nourizadeh , H. Namvar Arefi , M. T. Kheirkhah , Ind. J. Otolaryngol. Head Neck Surg. 2023, 75, 507. 10.1007/s12070-023-03600-z PMC10188841 37206834

[26] E. L. Huttlin , M. P. Jedrychowski , J. E. Elias , T. Goswami , R. Rad , S. A. Beausoleil , J. Villén , W. Haas , M. E. Sowa , S. P. Gygi , Cell 2010, 143, 1174. 21183079 10.1016/j.cell.2010.12.001 PMC3035969

[27] L. Lan , J. Li , Y. Chen , W. Chen , W. Li , F. Zhao , G. Chen , J. Liu , Y. Chen , Y. Li , C.‐D. Wang , Y. Zheng , Y. Cai , Hum. Brain Mapp. 2021, 42, 485. 33090584 10.1002/hbm.25238 PMC7776005

[28] J. V. Olsen , B. Blagoev , F. Gnad , B. Macek , C. Kumar , P. Mortensen , M. Mann , Cell 2006, 127, 635. 17081983 10.1016/j.cell.2006.09.026

[29] K. M. Igelstrom , T. W. Webb , M. S. A. Graziano , J. Neurosci. 2015, 35, 9432. 26109666 10.1523/JNEUROSCI.0551-15.2015 PMC6605196

[30] R. Salvi , B. D. Auerbach , C. Lau , Y. C. Chen , S. Manohar , X. Liu , D. Ding , G. D. Chen , Curr. Top. Behav. Neurosci. 2021, 51, 133. 32653998 10.1007/7854_2020_156

[31] R. Salvi , K. Radziwon , S. Manohar , B. Auerbach , D. Ding , X. Liu , C. Lau , Y.‐C. Chen , G.‐D. Chen , Am. J. Audiol. 2021, 30, 901. 33465315 10.1044/2020_AJA-20-00023 PMC9126116

[32] C. N. Hall , M. C. Klein‐Flugge , C. Howarth , D. Attwell , J. Neurosci. 2012, 32, 8940. 22745494 10.1523/JNEUROSCI.0026-12.2012 PMC3390246

[33] S. L. Williams , I. Valnot , P. Rustin , J.‐W. Taanman , J. Biol. Chem. 2004, 279, 7462. 14607829 10.1074/jbc.M309232200

[34] J. Zeng , G. Li , Y. Xia , F. Wang , Y. Wang , S. Xu , Y. Zhou , X. Liu , X. Xie , J. Zhang , Cancer Lett. 2020, 492, 185. 32758616 10.1016/j.canlet.2020.07.027

[35] I. Maurer , Neurobiol. Aging 2000, 21, 455. 10858595 10.1016/s0197-4580(00)00112-3

[36] P. J. Yao , E. Eren , E. J. Goetzl , D. Kapogiannis , Biomedicines 2021, 9, 1587. 34829816 10.3390/biomedicines9111587 PMC8615874

[37] T. Brozoski , B. Odintsov , C. Bauer , Front. Syst. Neurosci. 2012, 6, 9. 22383901 10.3389/fnsys.2012.00009 PMC3285819

[38] S. Xiong , Y. Song , J. Liu , Y. Du , Y. Ding , H. Wei , K. Bryan , F. Ma , L. Mao , Hear. Res. 2019, 375, 44. 30795964 10.1016/j.heares.2019.01.021

[39] C. M. Gray , P. König , A. K. Engel , W. Singer , Nature 1989, 338, 334. 2922061 10.1038/338334a0

[40] W. Singer , Neuron 1999, 24, 49. 10677026 10.1016/s0896-6273(00)80821-1

[41] W. Sedley , M. O. Cunningham , Front. Hum. Neurosci. 2013, 7, 595. 24065913 10.3389/fnhum.2013.00595 PMC3778316

[42] Y.‐C. Chen , S. Liu , H. Lv , F. Bo , Y. Feng , H. Chen , J.‐J. Xu , X. Yin , S. Wang , J.‐P. Gu , Front. Neurosci. 2018, 12, 9. 29410609 10.3389/fnins.2018.00009 PMC5787069

[43] A. M. Leaver , A. Seydell‐Greenwald , J. P. Rauschecker , Hear. Res. 2016, 334, 49. 26299843 10.1016/j.heares.2015.08.005 PMC7343340

[44] B. Vidal , M. Droguerre , L. Venet , M. Valdebenito , F. Mouthon , L. Zimmer , M. Charvériat , J. Neurosci. Methods 2021, 355, 109139. 33741345 10.1016/j.jneumeth.2021.109139

[45] G. F. Paxinos , K. Franklin , in The Mouse Brain in Stereotaxic Coordinates, Academic Press, Cambridge 2001.

[46] J. Sun , S. Han , L. Ma , H. Zhang , Z. Zhan , H. A. Aguilar , H. Zhang , K. Xiao , Y. Gu , Z. Gu , W. A. Tao , ACS Appl. Mater. Interfaces 2021, 13, 3622. 33443402 10.1021/acsami.0c19400

[47] H. Jia , D. Yu , Dev. Cogn. Neurosci. 2019, 39, 100687. 31377569 10.1016/j.dcn.2019.100687 PMC6969363

[48] R. D. Pascual‐Marqui , Methods Find. Exp. Clin. Pharmacol. 2002, 24D, 5. 12575463

[49] M. Fuchs , J. Kastner , M. Wagner , S. Hawes , J. S. Ebersole , Clin. Neurophysiol. 2002, 113, 702. 11976050 10.1016/s1388-2457(02)00030-5

[50] R. D. Pascual‐Marqui , arXiv. 2007, 11, 1455.

