# Supplementary material for: Functional Connectivity Alterations and Molecular Characterization of the Anterior Cingulate Cortex in Tinnitus Pathology without Hearing Loss
Source: Adv Sci (Weinh). 2023 Nov 27;11(3):2304709. doi: 10.1002/advs.202304709 (PMC10797451; doi:10.1002/advs.202304709)
Supplement: Supplementary file 1 — Supporting Information [file ADVS-11-2304709-s004.pdf]

ADVANCED SCIENCE
Open Access

Supporting Information

for *Adv. Sci.*, DOI 10.1002/advs.202304709

Functional Connectivity Alterations and Molecular Characterization of the Anterior Cingulate Cortex in Tinnitus Pathology without Hearing Loss

Ting Fan, Peng-Fei Guan, Xiao-Fang Zhong, Meng-Ya Xiang, Ying-Qiu Peng, Ruo-Qiao Zhou, Jia-Min Gong, Yu-Qing Zheng, A-Qiang Dai, Jia-Ling Feng, Hong-Zhe Yu, Jian Li, Hua-Wei Li* and Yun-Feng Wang**

Supporting Information

Functional Connectivity Alterations and Molecular Characterization of the Anterior Cingulate Cortex in Tinnitus Pathology without Hearing Loss

Ting Fan, Peng-Fei Guan, Xiao-Fang Zhong, Meng-Ya Xiang, Ying-Qiu Peng, Ruo-Qiao Zhou, Jia-Min Gong, Yu-Qing Zheng, A-Qiang Dai, Jia-Ling Feng, Hong-Zhe Yu, Jian Li, Hua-Wei Li *, Yun-Feng Wang**

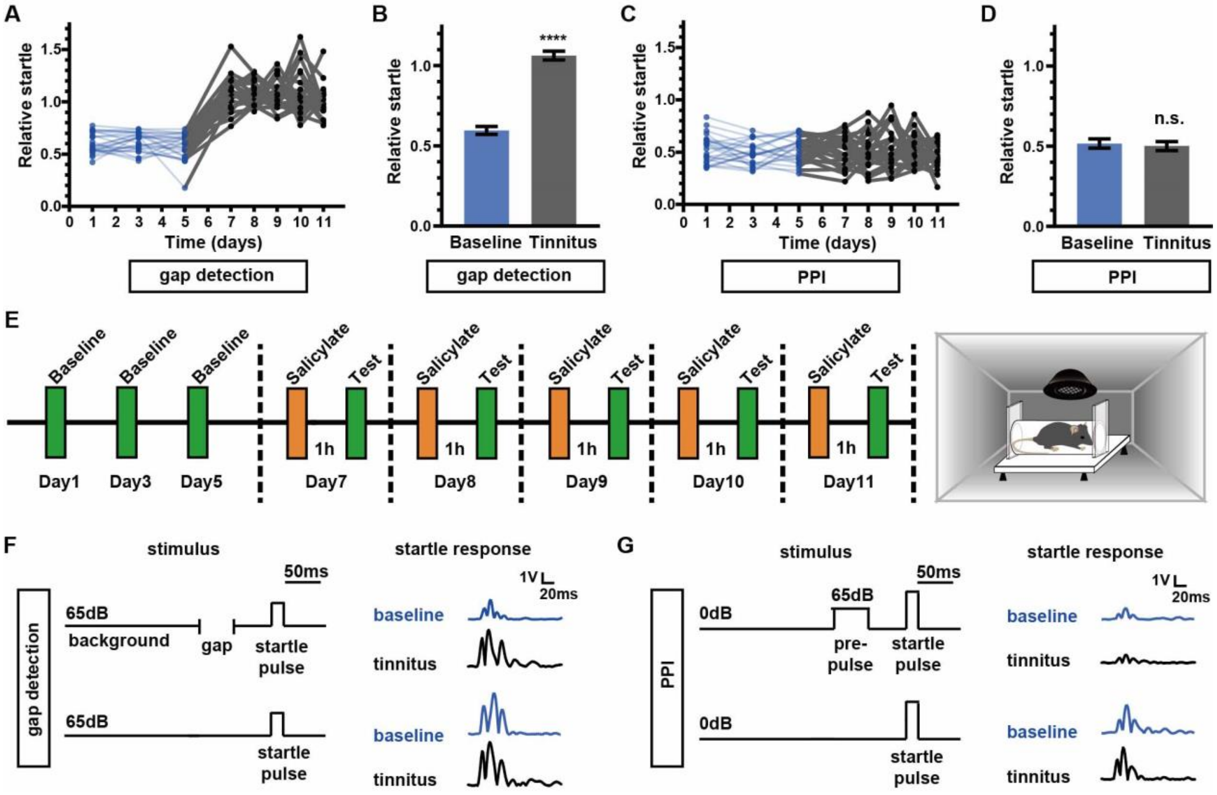

Figure S1. Behavioral evidence of tinnitus in mice.

(A) Relative startle of every individual mouse in gap detection. (B) Salicylate administration resulted in a significant deficit in gap detection compared with the baseline (C) Relative startle of every individual mouse in pre-pulse inhibition (PPI) session. (D) No significant difference was found in PPI during the whole timeline. Relative startle of gap detection (or PPI): a ratio of the mean amplitude in trials with silent gap (or pre-pulse stimulus) over the mean amplitude in trials without silent gap (or pre-pulse stimulus). Error bars indicate SD. Statistical difference of the relative startle between tinnitus and control groups was calculated by two-tailed *t*-test,

**** $p < 0.0001$, n.s. no significant difference. (E) Schematic timeline (left panel) and the experimental device (right panel) of the behavior testing. (F) Gap detection paradigm (left panel) and the typical waveform of startle response (right panel). (G) PPI paradigm (left panel) and the typical waveform of startle response (right panel).

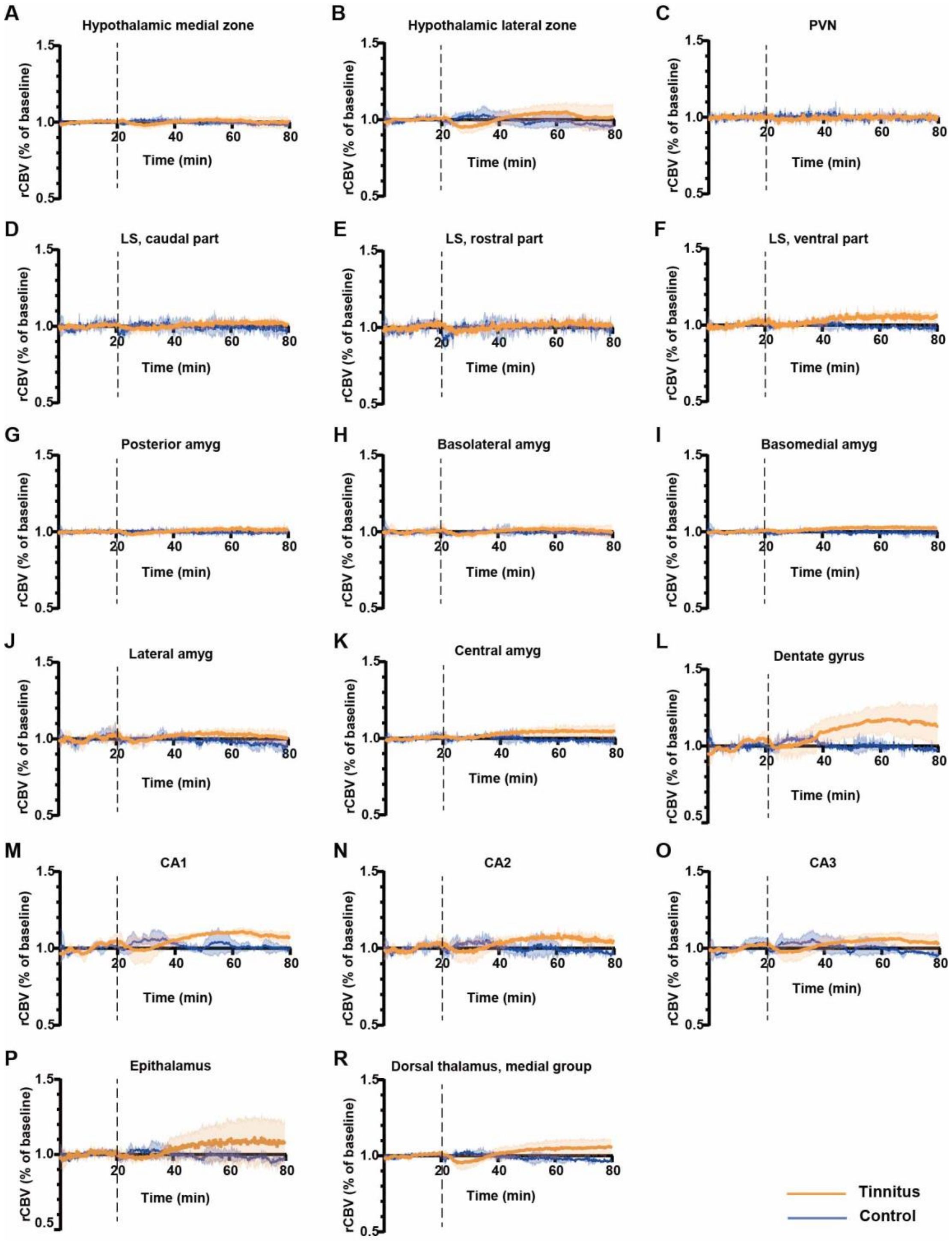

Figure S2. Relative cerebral blood volume (rCBV) changes of different brain areas in the limbic system in tinnitus (n=4) and control (n=3) mice.

PVN: paraventricular nucleus of hypothalamus; LS: Lateral septum; Amyg: Amygdala. The dashed line means injection time; CA1, CA2, CA3: cornu ammonis areas.

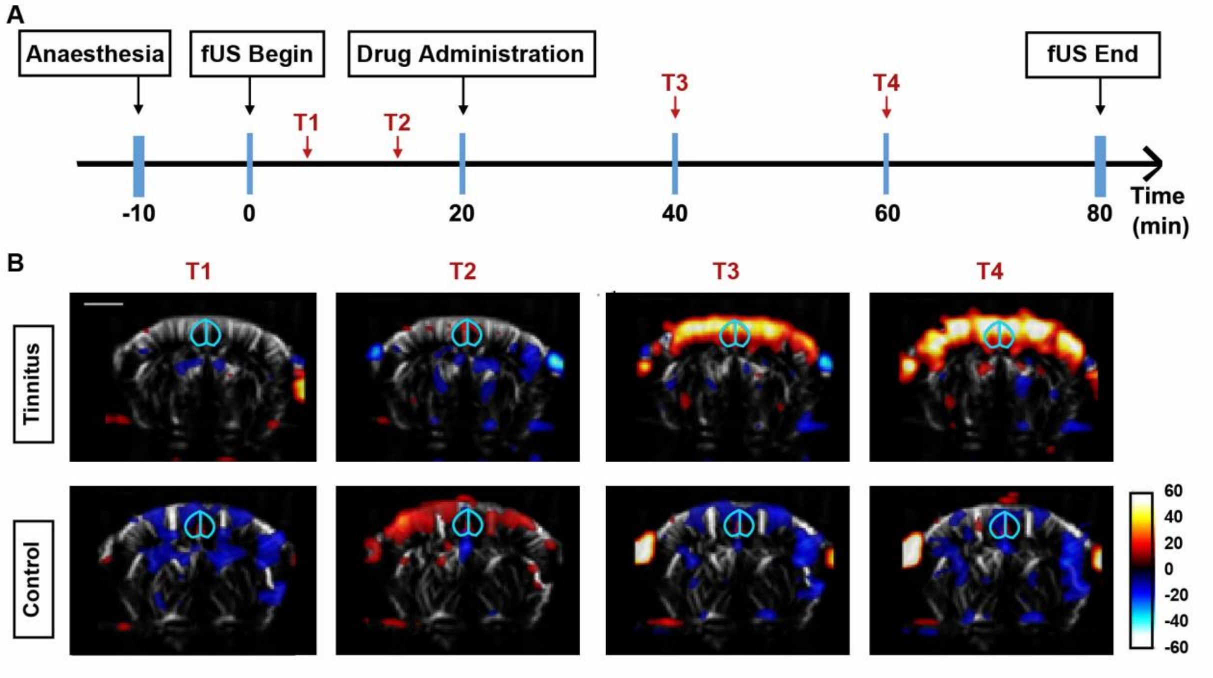

Figure S3. Representative brain activation maps of the anterior cingulate cortex in tinnitus (n=4) and control (n=3) mice.

(A) Flow chart of the functional ultrasound (fUS) experiment. (B) Representative functional ultrasound images of the anterior cingulate cortex in coronal slices of the mouse brain, outlined with light blue color and identified as corresponding to bregma 0.0 mm. The maps were depicted as the relative cerebral blood volume to the baseline level. Scale bar = 2 mm.

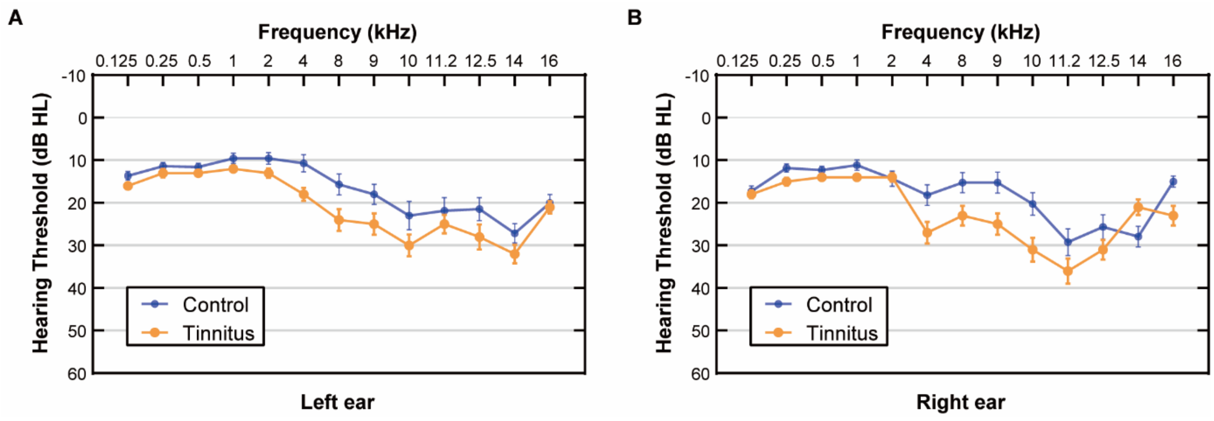

Figure S4. Pure tone hearing thresholds in tinnitus patients and healthy control subjects

for left (A) and right (B) ears.

The mean hearing thresholds (average hearing threshold at 0.5, 1, and 2 kHz) of both ears were less than 40 dB HL for all the tinnitus patients (n=64) and healthy control subjects (n=44). There was no significant difference in hearing thresholds of each frequency between the tinnitus group and control group calculated by two-tailed *t*-test.

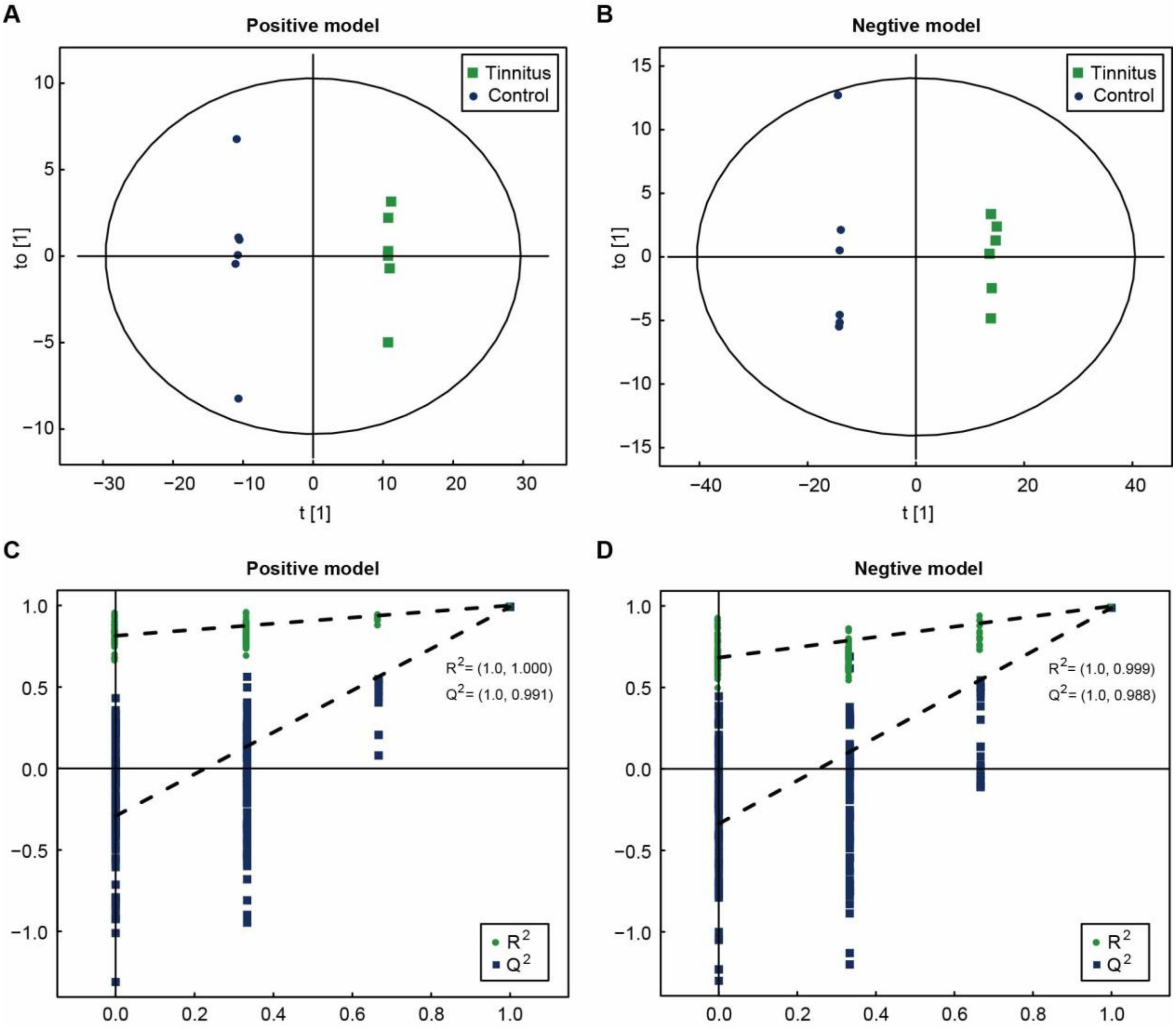

Figure S5. Score plots from OPLS-DA analysis and permutation tests.

(A) Score plots from OPLS-DA analysis of control group and tinnitus group; (B) Cross-validation plot of OPLS-DA mode of metabonomic data from the control and tinnitus. Anterior cingulate cortex tissue from 25 tinnitus mice and 28 healthy control mice were combined into 6 samples in each group.**Table S1. Increased functional connectivity in tinnitus patients compared with controls.**

Brain area (Brodmann area)	*pairs*	Brain area (Brodmann area)
primary somatosensory cortex 2 (BA2)		primary somatosensory cortex 1(BA1)
postcentral gyrus (BA 43)		primary somatosensory cortex 2 (BA2)
postcentral gyrus (BA 43)		primary somatosensory cortex 3 (BA3)
opercular part of inferior frontal gyrus (BA 44)		primary somatosensory cortex 3 (BA3)
postcentral gyrus (BA 43)		primary motor cortex (BA4)
opercular part of inferior frontal gyrus (BA 44)		primary motor cortex (BA4)
secondary auditory cortex (BA42)		dorsolateral prefrontal cortex (BA9)
opercular part of inferior frontal gyrus (BA 44)		superior temporal gyrus (BA22)
opercular part of inferior frontal gyrus (BA 44)		secondary auditory cortex (BA42)
insula (BA13)		postcentral gyrus (BA43)
primary auditory cortex (BA41)		pregenual anterior cingulate cortex (BA32)
postcentral gyrus (BA43)		pregenual anterior cingulate cortex (BA32)

Table S2. Tinnitus patient characteristics.

Variable	Overall (n=64)
Tinnitus side	
unilateral	40 (62.5%)
bilateral	24 (37.5%)
Tinnitus pitch	
high pitch	46 (71.9%)
low pitch	14 (21.9%)
noise-like tinnitus	4 (6.3%)
Duration of tinnitus (months)	21 (7.5, 36)
THI score	42 (30, 64)
VAS-L score	5 (4, 6)

VAS-A score	4 (3, 5)

Values are presented as median (P25, P75), or n (%).

THI: Tinnitus Handicap Inventory; VAS-L: Visual Analogue Scale-Loudness; VAS-A: Visual Analogue Scale- Annoyance.

Methods of Tinnitus Mice Model Establishment and Verification

A total of 60 mice were randomly divided into the control group and tinnitus group. The timeline of the tinnitus mice model establishment and verification were shown in the left panel of **Figure S1E**. After baseline behavioral testing (every other day for one week), mice in the tinnitus group were administered intraperitoneally with sodium salicylate (350mg/kg, Sigma-Aldrich, Saint Louis, MO) for 5 successive days as previously described, while mice in the control group were administered with the same amount of physiological saline. Behavioral tests were conducted 1h after drug injection.

Tinnitus was verified by the gap detection test (60 trials per session), before which the pre-pulse inhibition (PPI) was conducted as a control session (40 trials per session) at the same day. Both gap detection and PPI data were collected by startle reflex hardware and SR-LAB software (San Diego Instruments). Testing was done using wideband noise in a sound-attenuating chamber (**Figure S1E**, right panel). In the gap detection session, animals without tinnitus exhibit an inhibition of reflex to a startle stimulating sound (115 dB for 20 ms) when the constant background sound is presented with a silent gap (50 ms) embedded in it (**Figure S1F**). Animals that exhibit reduced ability to detect the silent gap background noise were considered mice with tinnitus. In the PPI session, testing was done in quiet background, and a pre-pulse stimulus (65 dB for 50 ms) was presented before the startle pulse (**Figure S1G**). Deficits in gap detection without any accompanied deficit in PPI suggest the presence of tinnitus rather than hearing loss or sensory gating dysfunction.[1] The criterion of a “tinnitus-positive” mice is as bellow: the relative startle of gap detection test is greater than 95% confidence interval (CI) of the mean baseline level.[2]

Processing Methods of the Mouse ACC Samples Before LC-MS/MS Analysis

After harvesting, the tissues of mouse ACC were frozen on liquid nitrogen and stored at -80℃ before metabolomics or proteomics. For metabolomics, samples were homogenized with 200uL

of H2O and five ceramic beads using the homogenizer, and the homogenized solution was used for metabolite extraction through adding 800uL methanol/acetonitrile (1:1, v/v) to it. Centrifugate the mixture for 20min (14000 g, 4 °C) and dry the supernatant in a vacuum centrifuge. For LC-MS analysis, we redissolved the sample in 100 μL acetonitrile/water (1:1, v/v) solvent and centrifuged it for 15 min at 14000 g at 4 °C, after which the supernatant was injected. For proteomics and phosphoproteomics, the ACC samples were lysed in SDT buffer (4%SDS, 100mM Tris-HCl, pH7.6) for protein extraction. Protein qualification and trypsin digestion were conducted as described before.[3,4,5] TiO2 beads were used to enrich the phosphorylated peptides.

References

[1] Turner, J., Larsen, D., Hughes, L., Moechars, D., and Shore, S. (2012). Time course of tinnitus development following noise exposure in mice. J Neurosci Res 90, 1480-1488.

[2] Park, S.Y., Kim, M.J., Park, J.M., and Park, S.N. (2020). A Mouse Model of Tinnitus Using Gap Prepulse Inhibition of the Acoustic Startle in an Accelerated Hearing Loss Strain. Otol Neurotol 41, e516-e525.

[3] Sun, J., Han, S., Ma, L., Zhang, H., Zhan, Z., Aguilar, H.A., Zhang, H., Xiao, K., Gu, Y., Gu, Z., et al. (2021). Synergistically Bifunctional Paramagnetic Separation Enables Efficient Isolation of Urine Extracellular Vesicles and Downstream Phosphoproteomic Analysis. ACS Appl Mater Interfaces 13, 3622-3630.

[4] Wiśniewski, J.R., Zougman, A., Nagaraj, N., and Mann, M. (2009). Universal sample preparation method for proteome analysis. Nat Methods 6, 359-362.

[5] Pi, S., Mao, L., Chen, J., Shi, H., Liu, Y., Guo, X., Li, Y., Zhou, L., He, H., Yu, C., et al. (2021). The P2RY12 receptor promotes VSMC-derived foam cell formation by inhibiting autophagy in advanced atherosclerosis. Autophagy 17, 980-1000.
